# Electrocardiographic imaging for cardiac arrhythmias and resynchronization therapy

**DOI:** 10.1093/europace/euaa165

**Published:** 2020-08-05

**Authors:** Helder Pereira, Steven Niederer, Christopher A Rinaldi

**Affiliations:** e1 Division of Imaging Sciences and Biomedical Engineering, King’s College London, 4th Floor, Lambeth Wing, St. Thomas’ Hospital, Westminster Bridge Rd, London SE1 7EH, UK; e2 Cardiac Physiology Services—Clinical Investigation Centre, Bupa Cromwell Hospital, London, UK; e3 Cardiovascular Department, Guys and St Thomas NHS Foundation Trust, London, UK

**Keywords:** Electrocardiographic imaging, Body surface electrocardiogram, Mapping, Arrhythmias, Assessment, Cardiac resynchronization therapy

## Abstract

Use of the 12-lead electrocardiogram (ECG) is fundamental for the assessment of heart disease, including arrhythmias, but cannot always reveal the underlying mechanism or the location of the arrhythmia origin. Electrocardiographic imaging (ECGi) is a non-invasive multi-lead ECG-type imaging tool that enhances conventional 12-lead ECG. Although it is an established technology, its continuous development has been shown to assist in arrhythmic activation mapping and provide insights into the mechanism of cardiac resynchronization therapy (CRT). This review addresses the validity, reliability, and overall feasibility of ECGi for use in a diverse range of arrhythmias. A systematic search limited to full-text human studies published in peer-reviewed journals was performed through Medline via PubMed, using various combinations of three key concepts: ECGi, arrhythmia, and CRT. A total of 456 studies were screened through titles and abstracts. Ultimately, 42 studies were included for literature review. Evidence to date suggests that ECGi can be used to provide diagnostic insights regarding the mechanistic basis of arrhythmias and the location of arrhythmia origin. Furthermore, ECGi can yield valuable information to guide therapeutic decision-making, including during CRT. Several studies have used ECGi as a diagnostic tool for atrial and ventricular arrhythmias. More recently, studies have tested the value of this technique in predicting outcomes of CRT. As a non-invasive method for assessing cardiovascular disease, particularly arrhythmias, ECGi represents a significant advancement over standard procedures in contemporary cardiology. Its full potential has yet to be fully explored.

## Introduction

Cardiovascular disease (CVD) is the leading cause of death in Western countries,^1^ and imposes a severe burden on society. While many advanced non-invasive measurement technologies have been developed, the 12-lead electrocardiogram (ECG) has been in use for over 100 years and has become established as the ‘de facto standard’ for the non-invasive assessment of a wide range of heart diseases. The ability to obtain numerical readings of electrical measurements of the heart has long helped healthcare professionals provide both prevention and treatment.

Despite being a powerful diagnostic tool, 12-lead ECG has some limitations when used to support management procedures in modern cardiology. The current pool of research has established that 12-lead ECG is limited in detecting the precise location of arrhythmias, or detailed electrical activation pattern when cardiac resynchronization therapy (CRT) is involved.^2^^,^^3^ Further drawbacks include its limited value in precisely identifying atrial and ventricular activation during arrhythmias, including accessory atrioventricular conduction activation,^4^ and in providing a detailed assessment of electrical ventricular activation abnormalities,^5^ inadequate pinpointing of the location of ventricular tachycardia (VT),^6^ and poor spatial resolution.^7^ These limitations may necessitate invasive electrophysiological studies to understand the patient’s underlying conditions.

Electrocardiographic imaging (ECGi) is a non-invasive, multi-lead ECG-type imaging tool (50 to approximately 300 electrodes depending on the manufacturer) that allows 3D visualization of the cardiac geometry with onset activation maps providing further diagnostic data compared to a conventional 12-lead ECG.^2^^,^^4^^,^^8^ It has been shown to be accurate for detecting a broad spectrum of specific arrhythmic characteristics.^3^ This novel method may be able to address the limitations of 12-lead ECG. This review addresses the validity, reliability, and overall feasibility of ECGi for use in a diverse range of arrhythmias.

## Search methodology

A systematic search through Medline via PubMed was performed through 19 January 2020, using various combinations of the following terms:

‘Arrhythmias, Cardiac’[MeSH]; ‘Arrhythmia, Sinus’[MeSH]; ‘Cardiac Resynchronization Therapy’[MeSH]; ‘Cardiac Resynchronization Therapy Devices’[MeSH]; ‘Body Surface Potential Mapping’[MeSH]; ‘Electrocardiographic Mapping’[All fields]; ‘Body Surface Electrocardiographic Mapping’[All fields]; ‘Electrocardiographic Imaging’[All fields]; ‘ECGi’[All fields].

The database search was performed using both American and British spellings. Throughout the process, the search was limited to full-text human studies published in peer-reviewed journals in the English language only. The article type was limited to ‘Clinical Study’, ‘Case Study’, ‘Case Report’, ‘Clinical Trial’, ‘Controlled Clinical Trial’, and ‘Randomized Controlled Trial’. No restrictions regarding the date of publication or sex or age of the participants were applied. Review articles, animal studies, *in vitro* or *in silico* experiments and simulations as well as studies focusing on other broader techniques were excluded from the review. Our initial intention was to develop a meta-analysis; regrettably it was soon evident that the identified studies used heterogeneous methodologies, particularly with regard to patient inclusion criteria, outcome assessment and comparison group. The primary focus was then changed to find and discuss studies that evaluated the application of ECGi in the context of patients with arrhythmia. For this purpose, we selected papers through manual screening of full texts that contained at least two of three key concepts: (i) arrhythmia, (ii) CRT, and (iii) ECGi.

In addition, all the references of the identified studies were screened using the same criteria to find further relevant publications that might have been missed during the initial search.

**Figure 1:**
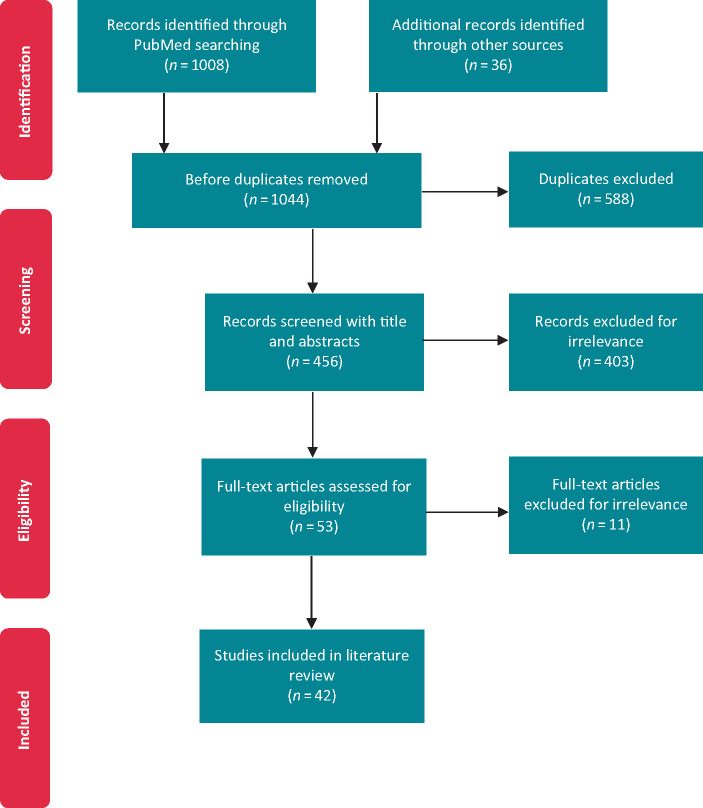
PRISMA diagram illustrating experienced stages of selection according to search and eligibility methodology.

The entire searching and screening processes were carried out by two independent specialists and decisions were made based on consensus. As a result, 42 articles were included for the final review, stages of selection are identified through the PRISMA diagram^9^ (*Figure 1*).

## Electrocardiographic imaging

**Figure 2:**
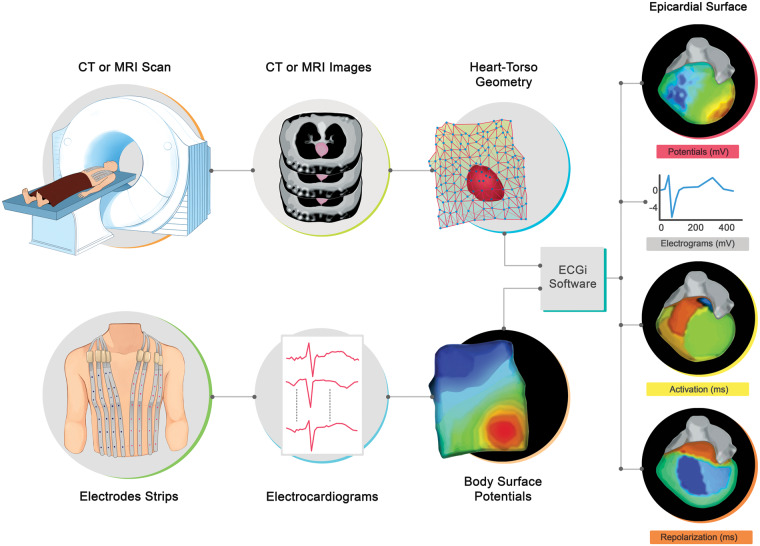
The ECGi procedure. Body surface potentials are recorded from several electrodes. The patient-specific heart-torso geometry is obtained from thoracic CT or MRI scan. The data are combined using mathematical algorithms to reconstruct epicardial potentials and unipolar electrograms on the heart surface. Maps of epicardial activation and recovery can be further derived from the electrograms. Adapted from Rudy *et al*.^10^ CT, computed tomography; ECGi, electrocardiographic imaging; MRI, magnetic resonance imaging. *Author's permission granted and RightsLink License number 4851570792908.*

Electrocardiographic imaging provides a mapping system that stores and displays electrophysiological data for analysis. The procedure consists of the patient wearing multiple electrodes (from 50 to close to 300, depending on the manufacturer), which can be contained within a vest or strips, connected to a mapping system, which records the electrical signals (*Figure 2*).

A computed tomography (CT) scan or magnetic resonance imaging (MRI) is used to display a 3D image of the heart (*Figure 2B*). During excitation, the heart creates an electrical potential field between the body surface and the epicardium. The potential field on the torso can be measured through electrodes on the torso. One of the functions of ECGi is to provide images of the instantaneous potential fields over time allowing a more complete view on the spatio-temporal^11^ epicardium data from measurements on the torso; an inverse problem of electrocardiography—which aims to recover noninvasively regional information about intracardiac electrical events from electrical measurements on the body surface.^12^ Apart from epicardial potentials, experimental validation studies^13^ have developed inverse electrocardiographic solutions through equivalent sources as single fixed-location dipole, a multipole series, moving dipoles, multiple fixed-location dipoles,^14^^,^^15^ homogeneous volume conductor,^16^ and activation wavefronts.^17^

Mathematically, it can be expressed through the following equation:^18^*ϕ_T_* = *Aϕ_E_*_,_ where *ϕ_T_* is the electric field on the torso surface and *ϕ_E_* is that on the epicardium. The information regarding the relation of the two surfaces (epicardium and torso) is expressed by the transfer matrix ‘*A*’.

Even small errors in the measurement of *ϕ_T_* (often resulting from electrode positioning) can be automatically reflected as a large error in the calculation of *ϕ_E_*. The most common mechanism used to control this inverse problem is Tikhonov regularization, a method that imposes constraints on *ϕ_E_*.^19^ Mathematically, this can be expressed as the vector that minimizes *ϕ_E_* by: min*ϕ_E_*[‖*ϕ_T_−A ϕ_E_*‖^2^ + *t*‖*Lϕ_E_*‖^2^], here *t* is a parameter of regularization and *L* is a regularization operator.

**Figure 3:**
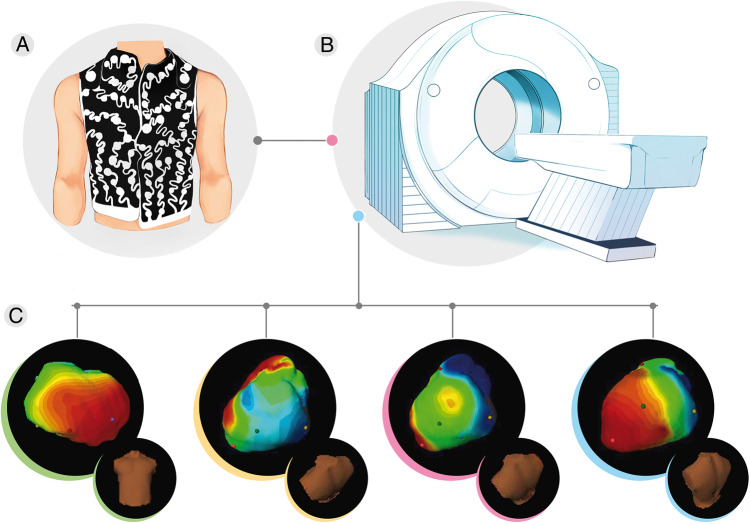
ECGi systems usually consist of a vest embedded with 50 to approximately 300 electrodes (depending on the manufacturer) that is fitted to the patient’s torso, which allows the acquisition of electrograms (*A*). Patients with the vest still in position will need to undergo a thoracic CT scan to determine the precise anatomic relation between the cardiac geometry and the torso electrodes (*B*). The ECGi system combines each data set obtained from the vest and CT scan, and an activation waveform map of both ventricles’ epicardial surface is generated and combined to construct 3D epicardial isochrone maps (*C*). CT, computed tomography; ECGi, electrocardiographic imaging.

With these algorithms, the ECGi system provides a 3D reconstructed image of the heart, from the information acquired from CT/MRI along with the location and electrical data of the electrodes (*Figure 3C*), as well as a beat-by-beat activation map,^6^ which gives an accurate onset of electrical activation. Tikhonov regularization and the generalized minimal residual algorithm are utilized throughout several steps such as heart and body-surface segmentation (image segmentation), creating the heart and torso geometric surface representation (generation of mesh), numerical algorithms and visualization.^20^

The ability to extract data non-invasively significantly enhances diagnosis. Based on a mathematical approach, ECGi is able to identify the electrical source for a given body-surface potential distribution. Identical body-surface potentials can appear due to various cardiac electrical activity patterns, most of which are unlikely to occur. In a clinical sense, ECGi surpasses the limitations of the current 12-lead ECG techniques because it provides a cardiac geometry where the onset of electric activity can be observed through activation maps of arrhythmias of the heart surface. Furthermore, standard ECG requires deciphering of body-surface data using cardiac activity under the assumption of a ‘standard’ heart shape and size in a ‘standard’ torso. In contrast, ECGi uses the precise heart-torso geometry of the patient to pinpoint the site of arrhythmia and locate the sequence on the heart.^21^

Research has shown that the ECGi mapping system can effectively deliver better information than conventional techniques, even for normal cardiac physiology.^18^ This is achieved because ECGi merges body-surface electrical data and the anatomical information from medical imaging to compute and process epicardial unipolar electrograms that are displayed through the epicardial surface of the heart.^22^ The method currently used for non-invasive diagnosis and detection of cardiac electrical activity involves a 12-lead ECG and is a routine part of medical care. However, ECG only measures cardiac electrical activity on the surface of the body and not on the heart itself. Thus, ECG is constrained by a spatial resolution that can determine regional electrical activity but cannot locate the regions where arrhythmic activities occur in the heart.^21^^,^^23^ The following text describes the development of ECGi over time, along with research that addresses different complex types of arrhythmias and illustrates how ECGi can deliver accurate information that can better inform an appropriate therapeutic approach.

## History of electrocardiographic imaging

The concept of ECGi, also referred to as electrocardiographic mapping, has been around for more than 40 years. Since the idea was first introduced in 1977,^24^ it has undergone many modifications. It is now being recognized as an advantageous diagnostic tool.

Initial studies on the concept were based on either computational or eccentric sphere models or equivalent cardiac electric generators.^25^ Most investigations that provided insight on the propagation of electrical activity were simulations in a membrane-based realistic-geometry computer models of the ventricles of the human heart.^26^

**Figure 4:**
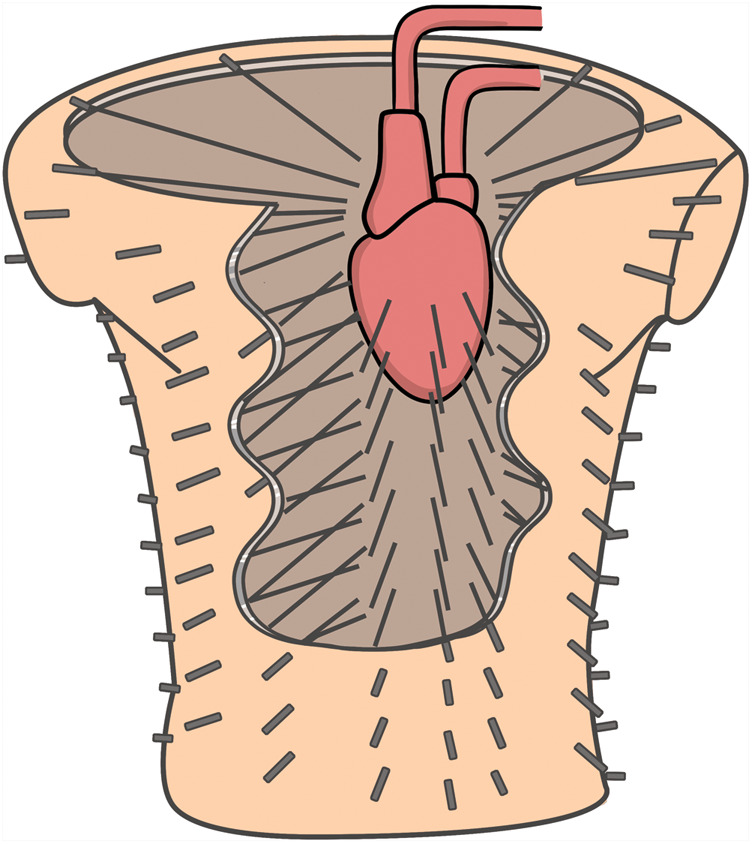
Torso tank with part of the anterior surface removed to reveal the heart and rods. Rods project from the surface of the body radially inward toward the central axis of the heart. Adapted from Oster *et al*.^18^*Author's permission granted.*

Extensive experiments on animal models investigated a wide range of physiological and pathological conditions. Most of these experiments were performed on large mammals, primarily dogs^27^ and pigs,^28^ to imitate the structure and relations of the heart inside the human body. One of the most important experimental setups was the torso tank (*Figure 4*).^18^ It consisted of a tank shaped like a human torso filled with an electrolyte solution. A perfused dog heart was placed inside the torso tank in a position that resembles the human anatomy. The system included 384 torso-surface electrodes and the same number of rods with electrodes at their tips placed inside the tank around the heart touching the epicardium.

The potentials, measured with these two sets of electrodes, were than compared to correlate the results and the positions of ‘invasive’ vs. ‘non-invasive’ electrodes. Oster *et al*.^18^ defined the basic principle for ECGi equipment and laid the groundwork for future investigations.

**Figure 5:**
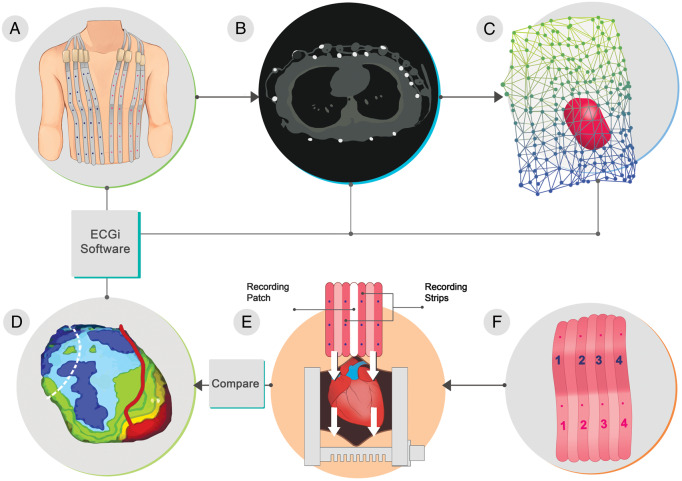
Study design used by Ghanem *et al*.^31^ ECGi in comparison to intraoperative mapping.17 (*A*) Vest. (*B*) Cross-sectional CT slice showing the heart and body-surface electrodes. (*C*) Subject-specific heart-torso geometry digitized from CT (circles indicate electrode positions). (*D*) Non-invasive epicardial potentials obtained from CT and body-surface potential mapping data using electrocardiographic imaging. (*E*) Illustration of the intraoperative mapping procedure showing the anterior where the epicardial patch is going to be inserted. (*F*) Epicardial patches used for intraoperative mapping. CT, computed tomography; ECGi, electrocardiographic imaging. *RightsLink License number 4851571240838*

The technology evolved over the years, mainly through simulation and model studies. Bidomain models of the cardiac tissue were used to investigate the propagation of excitation wavefronts and associated potential distribution and electrograms.^29^ Other studies used transmembrane voltages, epicardial potentials, and dipoles to validate ectopic origins through the inverse problem of electrocardiography.^30^ Ultimately, after the method was validated in animal *in vitro* and *in vivo* models, it was applied in humans. Early human studies included comparisons of images obtained using non-invasive ECGi to direct intra-operative mapping in open-heart surgery patients (*Figure 5*).^31^

Besides sinus rhythm, ventricular pacing with an external pacemaker was also recorded, which provided crucial information regarding the location of ectopic foci involved in the pathophysiology of various arrhythmias.

The latest major obstacle for ECGi research has been the lack of standards and unbiased comparisons of approaches and techniques in different centres. Papers have not always described all the components in sufficient detail to allow verification and comparison of the results. To overcome this problem, the Consortium for Electrocardiographic Imaging (CEI) was created in 2014.^32^ Its main goal is to provide researchers with unified and standardized frameworks for ECGi to facilitate collaboration and the validation of results.

## Clinical data on the use of electrocardiographic imaging

Several studies have used ECGi to establish its efficacy in clinical practice, mainly as a diagnostic tool for a variety of arrhythmias including atrial arrhythmias and ventricular arrhythmias, and for predicting outcomes of CRT(*Table 1*).

**Table 1:** Summary of studies

Author	Study type	Population	Intervention	Comparison	Conclusion
Knecht 2017^33^	Clinical study	118 patients with continuous AF duration <1 year	ECVUE™ 252 electrodes CardioInsight	None	Successful identification of biatrial AF drivers
Haissaguerre 2014^34^	Clinical study	103 consecutive patients with persistent AF	ECVUE™ 252 electrodes CardioInsight	12-lead ECG	ECGi detects AF driver domains and provides results similar to ECG of ablation with shorter radiofrequency delivery
Gage 2017^35^	Clinical study	66 patients undergoing CRT	ECGi 53 electrodes Medtronic	None	Potential improvement in selection and optimization of patients receiving CRT
Shah 2013^36^	Clinical study	52 patients with ATs	ECGi 252 electrodes CardioInsight	CARTO or NavX	Successful and accurate identification of mechanism and location of AT
Erkapic 2015^37^	RCT	42 patients with monomorphic PVCs with or without monomorphic VT	ECVUE™ 252 electrodes CardioInsight	12-lead ECG algorithms	Precise VA mapping and more targeted ablation with higher radiation exposure due to CT
Johnson 2017^38^	Clinical study	40 CRT-indicated patients	53-electrode body-surface mapping Heartscape Technologies	Catheter-based assessment	Better identification of haemodynamically optimal sites for lead location
Ploux 2013^39^	Clinical study	33 CRT candidates with LBBB and non-specific intraventricular conduction disturbance	ECVUE™ 252 electrodes CardioInsight	12-lead ECG	ECGi provides ventricular electrical uncoupling that can provide improved prediction of CRT clinical response
Zhang 2015^40^	Clinical study	31 patients: 25 patients with BrS and 6 patients with RBBB	ECGi 250 electrodes CardioInsight	None	ECGi could differentiate BrS from RBBB
Revishvili 2015^41^	Clinical study	29 patients with cardiomyopathy and implanted CRT	NEEES 224 electrodes EP Solutions SA	CARTO 3	Correct highly accurate identification of the pacing site from various endo- and epicardial sites
Cakulev 2013^4^	Clinical study	27 patients with WPW, PVC, AT, AF	ECVUE™ 250 electrodes CardioInsight	Clinical Diagnoses	Valid activation sequence mapping in different types of arrhythmias
Cochet 2014^2^	Clinical study	27 patients with VT, WPW, AF, VF	ECGi 252 electrodes CardioInsight	None	ECGi may contribute to a more comprehensive assessment of various types of arrhythmias, improving diagnosis, therapy, and prognosis
Ghosh 2011^42^	Clinical study	25 non-ischaemic cardiomyopathy patients who were previously implanted with a CRT device	ECGi 250 electrodes CardioInsight	None	Through ED, ECGi characterizes different important patterns of CRT responders in native and synchronized periods
Wang 2011^21^	Clinical study	25 patients undergoing catheter ablation procedures for various forms of VT with or without PVCs	ECGi 256 electrodes BioSemi	catheter-based electrophysiology mapping	Accurate mapping of the VT activation sequence, depth of origin, and location
Vijayakumar 2014^43^	Clinical study	25 patients with genotype- and phenotype-positive LQTS	ECGi 256 electrodes CardioInsight	None	ECGi reveals important characteristics in LQTS patients, which are not detected by conventional ECG.
Jamil-Copley 2014^6^	Clinical study	24 patients with OTVT/PVC	ECGi 252 electrodes CardioInsight	12-lead-ECG algorithms	Catheter ablation can be improved with ECM that accurately determines the origin of OTVT and PVC
Cuculich 2011^44^	Clinical study	24 patients with VT and history of MI	ECGi 256 electrodes BioSemi	None	Successful identification of areas of anatomic scar with ECGi
Wissner 2017^45^	Clinical study	20 patients with monomorphic PVCs or VT	NEEES 224 electrodes	CARTO 3	Accurate identification of the PVC/VT site
Andrews 2017^46^	Clinical study	20 genotyped ARVC patients with a broad spectrum of disease	ECGi 256 electrodes ActiveTwo BioSemi	None	ECGi provides characteristic properties of electro-physiological substrate in ARVC patients
Berger 2011^47^	Clinical study	20 patients 10 patients with CHF undergoing CRT and 10 patients without structural heart disease	NICE 65 electrodes	None	NICE allows the visualization of endocardial and epicardial ventricular activation patterns, which can help to identify CRT responders with improved lead placement
Misra 2018^48^	Clinical study	20 patients presenting for catheter ablation of VT or PVC	ECGi 12-lead ECG VIVO	CARTO	Earliest activation of VA
Ghosh 2008^49^	Clinical study	14 paediatric patients with WPW syndrome and no other congenital disease	ECGi 200 electrodes CardioInsight	12-lead ECG-based algorithm	Successful localization of ventricular insertion sites of accessory pathways and evaluation of the outcomes
Yu 2018^50^	Clinical study	13 patients with PVC	ECGi 208 electrodes BioSemi	CARTO 3	Excellent performance in localizing the initial sites of focal VA
Varma 2015^51^	Clinical study	11 patients with HF and LBBB after CRT implantation	ECGi	None	ECGi may lead to a more personalized approach and enhancement of CRT response
Potyagaylo 2019^52^	Clinical study	10 patients with previously implanted CRT devices	ECGi 240 electrodes FRA + DTW	FRA	Reduced localization error and a significant accuracy improvement for clinical data of CRT patients with a complex aetiology
Silva 2009^53^	Clinical study	8 paediatric heart-failure patients with CHD undergoing evaluation for CRT	ECGi 250 electrodes CardioInsight	None	ECGi was used to assess ventricular ED and identify candidates for CRT among paediatric patients and help with correct lead placement
Jia 2006^54^	Clinical study	8 patients undergoing CRT during native rhythm and various pacing modes	ECGi 224-electrodes CardioInsight	None	ECGi provided important insights reflecting underlying pathologies
Tsyganov 2018^8^	Clinical study	8 patients with ischemic and non-ischemic cardiomyopathy and inducible ventricular arrhythmias	NEEES 224 electrodes	None	ECGi successfully visualizes macro-re-entrant circuits in scar-related VT
Berger 2006^55^	Clinical study	7 patients with WPW syndrome undergoing catheter ablation of the accessory pathway	NICE 65 electrodes	CARTO	NICE localized the sites of origin of ventricular pre-excitation in patients with WPW syndrome, similar to CARTO
van Dam 2013^56^	Clinical study	7 patients with PVCs	ECGi 12 lead ECG	None	Accurate PVC localization
Cuculich 2010^23^	Clinical study	6 patients with a history of AF	ECGi 256 electrodes CardioInsight	CARTO	Effective and patient-specific mapping of epicardial activation patterns in patients with AF
Cuculich 2017^57^	Clinical study	5 patients with high-risk, refractory VT undergoing SBRT	ECGi 256 electrodes BioSemi	None	ECGi can improve radioablation therapy and reduce the burden of VT
Wang 2018^58^	Clinical study	4 patients with scar-related VT	ECGi 120 electrodes	None	ECGi mapping may provide improved information about arrhythmogenic substrates and better visualization of 3D re-entry circuits
Sapp 2012^59^	Clinical study	4 patients undergoing epicardial catheter mapping and ablation of VT	ECGi 120 electrodes	None	Accurate identification of epicardial VT pacing sites and ventricular activation sequences
Wang 2011^60^	Clinical study	4 patients referred for ablation associated with myocardial infarction	ECGi 120 electrodes	CARTO	Accurate quantification of the scar substrate and capturing abnormal EP patterns
van Dam 2016^61^	Clinical study	3 patients with symptomatic idiopathic PVCs	ECGi 12-lead ECG	None	Correct identification of the PVC origin
van Dam 2009^62^	Clinical study	3 patients: 1 with WPW, 1 with BrS, and 1 healthy subject	ECGi 64 electrodes	None	The inversely estimated timing agreed with available physiological knowledge
Wang 2016^63^	Clinical study	2 patients undergoing catheter ablation of scar-related VT	ECGi 120 electrodes	None	Potential improvement of the spatial construct of a re-entry circuit
Intini 2005^64^	Case report	An athlete with focal VT	ECGi 224 electrodes	None	ECGi mapping guidance of the diagnosis and therapy of VT
Haissaguerre 2013^65^	Case report	A patient with persistent AF for 7 months	ECGi 252 electrodes CardioInsight	None	Successful identification of active sources, such as rotors and PV foci, validated by ablation results
Zhang 2013^66^	Case report	A patient with non-ischaemic cardiomyopathy, reduced LV, and VT	ECGi 252 electrodes CardioInsight	None	ECGi can successfully guide the diagnosis and treatment of VT
Schulze 2017^67^	Case report	A patient with a sustained monomorphic VT	ECGi 51 electrodes BioSemi	None	ECGi determines activation times of ventricular electric activity

AF, atrial fibrillation; ARVC, arrhythmogenic right ventricular cardiomyopathy; AT, atrial tachycardia; BrS, Brugada syndrome; CHD, congenital heart disease; CHF, congestive heart failure; CRT, cardiac resynchronization therapy; CT, computed tomography; DTW, dynamic time wrapping; ECG, electrocardiography; ECGi, electrocardiographic imaging; FRA, fastest route algorithm; HF, heart failure; LBBB, left bundle branch block; LQTS, long QT syndrome; MI, myocardial infarction; NEEES, non-invasive epicardial and endocardial electrophysiology system; NICE, non-invasive imaging of cardiac electrophysiology; OTVT, outflow tract ventricular tachycardia; PVC, premature ventricular complexes; RBBB, right bundle branch block; RCT, randomized controlled trial; SBRT, stereotactic body radiation therapy; VF, ventricular fibrillation; VIVO, view into ventricular onset; VT, ventricular tachycardia; WPW, Wolf–Parkinson–White syndrome.

## Atrial arrhythmias

**Figure 6:**
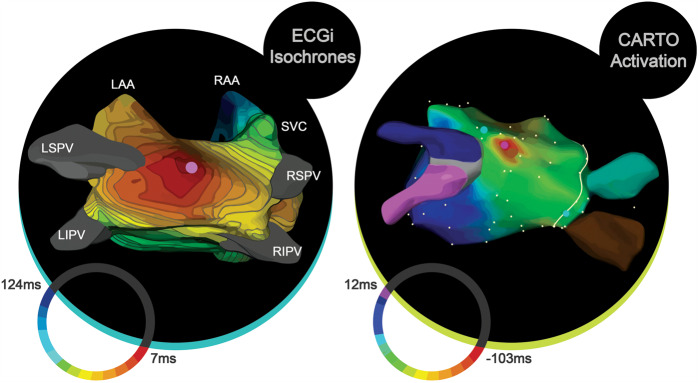
Adapted from Cakulev *et al*.^4^ On the left, ECGi map demonstrated focal activation of the left atrium. On the right, CARTO map shows the site of successful ablation, which matched the site of earliest activation through ECGi. ECGi, electrocardiographic imaging; LAA, left atrial appendage; LIPV, left inferior pulmonary vein; LSPV, left superior pulmonary vein; RAA, right atrial appendage; RSPV, right superior pulmonary vein; RIPV, right inferior pulmonary vein; and SVC, superior vena cava. *Author's permission granted.*

Five studies have examined ECGi and its role in assisting the diagnosis of atrial arrhythmias compared to standard 12-lead ECG.^2^^,^^4^^,^^23^^,^^36^^,^^65^ Two of the studies examined participants with atrial tachycardia (AT);^4^^,^^36^ while the other three examined participants with atrial fibrillation (AF).^2^^,^^23^^,^^65^ All five studies obtained 3D mapping sequences to assess the accuracy in comparison to invasive ablation procedures (*Figure 6*).

In the two studies that examined participants with AT, one (*N = *52) reported a nearly complete agreement between the results of ECGi and invasive procedures for detecting the site of arrhythmia in 48 participants,^36^ while the other (*N = *10) reported a 100% success rate in the localization of atrial arrhythmia.^4^ Shah *et al*.^36^ reported a diagnostic accuracy of 85% for re-entrant ATs (23 of 27 cases) and 100% for focal ATs (21 cases), which represented an average accuracy of 92% (44 of 48 cases). Additionally, the accuracy was inferior for previously ablated atria (83%; 19 of 23 cases) compared to un-ablated atria (100%; 25 of 25 cases). Electrocardiographic imaging accurately identified all focal ATs as originating in the right atrium or left atrium. Further evaluation compared the diagnostic accuracy of ECGi between participants who had undergone previous AF ablation and those who were undergoing AF ablation for the first time.

In a study from 2010, Cuculich *et al*.^23^ further demonstrated that ECGi could be used as an alternative non-invasive mapping tool to analyse mechanisms of AF in humans. Electrocardiographic imaging was shown to image low-amplitude signals of AF in patients with various clinical characteristics (wavelets and focal sites) with an accuracy of 97%.^23^ The non-invasive mapping of epicardial activation patterns was successfully utilized to correctly identify locations critical to the maintenance of AF, ablation of which restored sinus rhythms with no further complications.^23^^,^^57^ Ablation guided by ECGi converted AF into AT in one patient, whose follow-up showed no symptoms or need for AF-associated medication.^65^

Electrocardiographic imaging gave an accurate diagnosis 92% of the time, with accuracies of 83% and 100% in the previous and first-time ablation groups, respectively. Cakulev *et al*.^4^ reported similar findings. They found that the use of ECGi assisted in the accurate identification of and discrimination between left and right atrium focal sources and re-entrant mechanisms in all participants.

A report by Cochet *et al*.^2^ in AF patients described the successful detection of rotor activity on the epicardium and accurate detection of the core location of trajectories. Moreover, ECGi made it possible to map epicardial activation patterns specific to the patient, which provides insight into AF mechanisms and demonstrates its importance as a non-invasive technique.^23^^,^^65^ It is evident from the studies described above that ECGi has strong diagnostic capabilities, consistent with a reliable and valid assessment method. As a result of recent advances, ECGi can measure and accurately depict atrial activation sites, rendered by a mapping system and consisting of beat-by-beat tracking. In addition, ECGi has enhanced the ability to determine whether atrial arrhythmia is focal or re-entrant, and to localize its origin to the left or right atrium. These studies show that ECGi reduces the need for further invasive procedures and can analyse AF patterns in a real-world setting over longer periods from minutes to hours.^57^

Electrocardiographic imaging can also identify AF driver domains (either focal or re-entry) along with their cumulative density map. In a study of 103 patients with persistent AF, Haissaguerre *et al*.^34^ targeted ablation to the driver domains detected by ECGi. Compared to the conventional approach (involving pulmonary vein isolation-electrogram-based ablation lines), the investigators found similar termination rates at 12 months, but reduced mean radiofrequency delivery to AF termination with driver domain identification (*P* < 0.0001). In a similar study, a group of 108 patients with persistent AF from eight European centres underwent ECGi before ablation to detect AF drivers. Several sites were ablated if required. After 1 year of follow-up, 78% and 77% of patients were off antiarrhythmic medications and free from AF recurrence, respectively. Nonetheless, a significant percentage of patients experienced AT recurrence that warranted further treatment.^33^

## Ventricular arrhythmias

The effectiveness of ECGi also extends to ventricular arrhythmias, specifically VT, which is heterogeneous in nature. The location of activation sites in VT is a particular challenge in practice.^21^ As with atrial arrhythmias, several studies have investigated the role of ECGi in accurately providing mapping sequences of ventricular activation sites.

Among the studies described below, three included participants with Wolf–Parkinson–White (WPW) syndrome,^2^^,^^4^^,^^62^ nine included patients with premature ventricular complexes (PVC),^4^^,^^6^^,^^21^^,^^37^^,^^45^^,^^48^^,^^50^^,^^56^^,^^61^ and one compared the ability of QRS duration and left bundle branch block (LBBB) to ECGi for predicting CRT outcomes.^39^ The studies that examined patients with WPW syndrome approached the disease mechanism in different ways. One study used ECGi to predict if the ventricular origin was localized to the right or left side of the ventricular septum,^4^ while the others used ECGi to predict the localization of essential accessory pathways and sites of ventricular pre-excitation.^2^^,^^62^ In both studies, ECGi accurately predicted the mechanisms. Thus, ECGi could provide unique critical details that would be unavailable by other tools.

Several studies have validated the use of ECGi mapping for catheter ablation in patients with PVC/VT.^21^^,^^50^^,^^59^^,^^63^ The localization of the VT and PVC pacing sites by non-invasive electrophysiological mapping using 120^21^^,^^59^^,^^63^ or up to 208 body surface electrodes^50^ prior to the invasive procedure was shown to be correlated with the results from the invasive mapping system CARTO.

Electrocardiographic imaging can also be clinically useful in cases of VT, where, by providing high spatial resolution and images of the activation sequences over the entire ventricles, it helps us better understand the processes of VT initiation and continuation. This further might give insights regarding relationships to an abnormal electrophysiological substrate or anatomical scars.^21^ Three case reports demonstrated the successful application of ECGi in guiding the diagnosis and therapy of VT by accurately localizing pathologic foci.^64^^,^^66^^,^^67^

**Figure 7:**
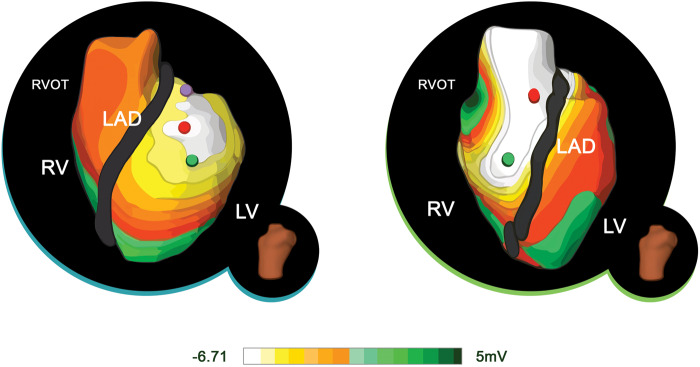
ECGi potential maps of RVOT and LVOT ectopy. The images show the ECGi potential PVC map from LAO views. On the left, early activation sequence favours LV. (*B*) On the right, early activation sequence favours RV. ECGi, electrocardiographic imaging; LAD, left anterior descending; LV, left ventricle; RV, right ventricle; RVOT, right ventricular outflow tract. Adapted from Jamil-Copley *et al*.^6^*Author's permission granted and RightsLink License number 4851580447554.*

Most of the studies that investigated PVCs sought to address the ability of ECGi to localize the origin of the PVC to the specific ventricular chamber (*Figure 7*). Jamil-Copley *et al*.^6^ reported that ECGi could predict the chamber of origin in 96% of the participants (23 of 24) and was 100% accurate at predicting localization in specific anatomical regions within the specified chamber. Compared to the results with ECGi, which were near-perfect, the three algorithms together predicted the correct location (i.e. chamber) of PVC in only 50–80% of the instances. Moreover, when the proper chamber was located, the specific region within the chamber was predicted only 37–58% of the time. The ability to correctly distinguish between chambers suggests that ECGi may be useful as a guide during invasive procedures, such as catheter ablation.^6^

To date, only one randomized controlled trial has evaluated a novel ECGi technology called ECVUE™ (Medtronic, Dublin, Ireland), which showed 95.2% accuracy in identifying both the chamber and origin of VA compared to conventional 12-lead ECG algorithms (76.2% and 38.1%, respectively). ECVUE™ also led to a shorter time to ablation and a lower number of radiofrequency-energy applications.^37^ Similar results were observed in another clinical study by Wissner *et al*.^45^ with 95% and 86% precision of chamber and ventricular segment detection, respectively. In both investigations, patients experienced 95% ablation success.^37^^,^^45^

In a study of four patients, Wang *et al*.^58^ showed that ECGi can be clinically feasible for scar-related VT by identifying the sites of myocardial scar and signal fractionation. The authors demonstrated that, in programmed induced VT, simultaneous epi-endo ECGi might be used for mapping scar substrates and re-entry circuits. Another investigation by Tsyganov *et al*.^8^ showed that ECGi was able to detect various patterns of induced VT. Electrocardiographic imaging visualized macro-re-entrant circuits in patients with ischaemic cardiomyopathy (*n* = 3) and cardiac sarcoidosis (*n* = 1), and relatively stable rotor activity and multiple wavelets in patients with hypertrophic cardiomyopathy (*n* = 1), Brugada syndrome (BrS) (*n* = 2), and idiopathic ventricular fibrillation (VF) (*n* = 1).

The number of body surface leads varies depending on the manufacturer. However, some investigators have reported the successful application of simplified ECGi that utilized standard 12-lead ECG. In this model, images obtained from MRI or CT specify the correct placement of ECG electrodes for electrophysiological mapping.

In three small studies, van Dam *et al*.^56^^,^^61^^,^^68^ were able to precisely localize the site of the PVC origin in patients undergoing catheter ablation with the help of electrophysiological mapping that utilized the inputs from 12-lead ECGs and MRI. Interestingly, this approach wasn’t limited to the epicardial surface, as in many cases, but was able to quantitatively localize PVCs on the ventricular walls, endocardium and intra-myocardium,^56^^,^^68^ or even to papillary muscles.^61^

In another investigation, a similar system called View into Ventricular Onset (VIVO) was shown to accurately predict earliest activation and foci of VA in 19 of 22 analysed patients with PVC or VT undergoing catheter ablation.^48^

## Arrhythmogenic syndromes

Electrocardiographic imaging might provide further insights in other arrhythmogenic syndromes. In a study of 25 patients with BrS, Zhang *et al*.^40^ showed that the abnormal electrophysiological substrate was localized in the right ventricular outflow tract that displays delayed activation, prolonged repolarization, and steep repolarization gradients. Six patients with right bundle branch block (RBBB) and seven other healthy individuals served as comparison and control groups, in which the characteristics seen in BrS were absent. By revealing the differences in epicardial activation, repolarization, and electrogram morphologies, ECGi might help to differentiate BrS from RBBB.

A different study used ECGi to map the cardiac electrophysiological substrate in 25 patients with genetically and phenotypically established long QT syndrome (LQTS). With some variations between and within genotypic groups, one of the characteristics of the electrophysiological substrate in LQTS appeared to be the regions of steep spatial dispersion of repolarization in the ventricular epicardium as well as a long activation–recovery interval. These findings further supported the previous theory of re-entry as a main mechanism in LQTS. The authors concluded that ECGi might play a potential role in risk stratification in patients with this disorder.^43^

Electrocardiographic imaging can be of clinical significance in WPW syndrome. In 14 children diagnosed with WPW syndrome without other cardiac abnormalities, ECGi mapping correctly localized and evaluated the ventricular insertion sites of accessory pathways for further ablation guidance.^49^ Berger *et al*.^55^ also reported the accurate detection of accessory pathway insertion sites in a study of seven adult patients with WPW syndrome using ECGi.

Arrhythmogenic right ventricular cardiomyopathy (ARVC) is a clinical disorder characterized by RV dilation, RV dysfunction, and regional RV wall motion abnormalities. Impairment in desmosome function leads to myocardial injury that causes fibrofatty infiltration in the RV and some parts of the left ventricle. Patients with ARVC are at significant risk of developing VT and sudden cardiac death. Andrews *et al*.^46^ suggested that ECGi may provide a better understanding of the disease substrate and improve patient care. In their study, the investigators evaluated 20 patients with ARVC and 20 healthy individuals. The authors found longer ventricular activation duration and prolonged mean epicardial activation-recovery intervals, all of which co-localized with pathological anatomical scar detected by cardiac MRI.

## Cardiac resynchronization therapy response

**Figure 8:**
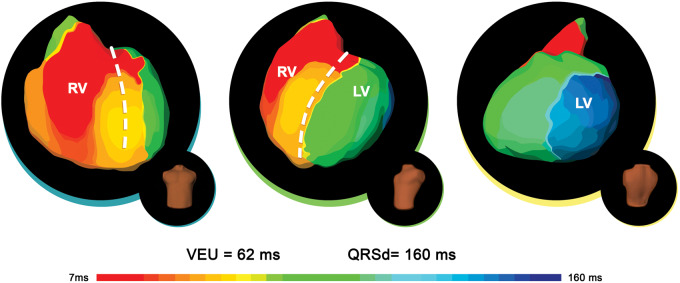
ECGi activation maps of a clinical responder to cardiac resynchronization therapy. Epicardial ventricular surfaces are displayed in three views (from left to right): anteroposterior, left anterior oblique, and posterolateral. The right ventricular lateral breakthrough is followed by rapid activation of the RV. The wave front spreads to the LV, with a first base-to-apex line of slow conduction (crowding of isochrones). LV activation ends at the lateral base. Adapted from Eschalier *et al*.^69^ ECGi, electrocardiographic imaging; LV, left ventricle; RV, right ventricle.*Author's permission granted and RightsLink License number 4851580660709*.

Electrocardiographic imaging may also play an important role in CRT. As an example, in patients with already-implanted CRTs, ECGi may aid in finding the pacing site with high accuracy, as demonstrated in a study of 29 patients by Revishvili *et al*.^41^ Ploux *et al*.^39^ aimed to evaluate the effectiveness of ECGi for predicting successful outcomes when applied to CRT compared to QRS duration. This was achieved by measuring ventricular activation time in patients with QRS of 120 ms or more. They observed that the use of ECGi to establish ventricular electrical uncoupling (VEU), which is the difference between left ventricular activation time and RV activation time, provides a much more accurate picture of the CRT response than 12-lead ECG predictions of QRS duration. Jia *et al*.^54^ also noted that ECGi can play a useful role by providing better insight into electrical synchrony and ventricular activation times, especially regarding the CRT response (*Figure 8*).

It is still a challenge to understand why some patients are more likely to benefit from CRT than others. Innovative research is underway, focusing on intraventricular delay patterns, indexes of QRS prolongation and ventricular activation dyssynchrony, to provide some insight into this issue. In light of the capacity of ECGi to provide mechanistic insights for arrhythmias, it may be able to provide detailed information about the therapeutic response. Principally, ECGi can provide information that can be used to determine whether CRT would benefit specific patients.

The ability to predict whether a patient will benefit from CRT is a valuable asset. Silva *et al*.^53^ demonstrated why accurate data are needed to predict CRT outcomes. In their study, the participants consisted of paediatric patients with heart failure who were in the process of obtaining some form of CRT. The mechanism studied was ventricular electrical dyssynchrony (ED), which is typically 20 ± 4 ms. When the subjects were divided into responders and non-responders to CRT, the study confirmed that the responder group had an ED index of 22 ms, while non-responders had an ED index of 37 ms. Thus, ECGi was able to provide data for determining which patients would benefit from CRT. These findings were further supported by another study where, in a group of 25 patients with non-ischaemic cardiomyopathy who had been previously implanted with a CRT device, ECGi, when performed during different programmed pacing, could accurately characterize CRT responders. During intrinsic rhythm, CRT responders showed very high dyssynchrony (ED = 35.5 ± 3.9 ms), which was significantly lowered (ED = 23.2 ± 4.4 ms) during optimal modes of CRT.^42^ One final advantage of ECGi is its ability to guide lead placement during implantation. The mapping system can accurately pinpoint electrical activation sites and provide details on the location of the last excitation area.^53^ This was shown in a study by Berger *et al*.^47^ on patients with congestive heart failure undergoing CRT; ECGi can be helpful in identifying responders to CRT. By providing adequate visualization of both endocardial and epicardial ventricular activation, it may lead to patient-specific lead placement and improved device-programming.

Electrocardiographic imaging was used to assess the extent to which right ventricular pacing (RVP) in biventricular pacing leads to left ventricular activation during CRT. In 11 CRT patients with left LBBB, RVP was shown to have different effects on LBBB-induced conduction problems. This finding may change the perception of RVP having LBBB-like effects, which led to the avoidance of RVP during biventricular stimulation.^51^

Electrocardiographic imaging may be more specific, accurate and sensitive than 12-lead ECG in the assessment, diagnosis and management of cardiac arrhythmias.^36^ The studies discussed above demonstrate that ECGi is a more insightful technology for the localization of arrhythmias, which once relied solely on 12-lead ECG. This is justified due to the higher validity and reliability shown by ECGi in a wide range of studies and in multiple patient groups. The mapping system has been used to assess various arrhythmias including AF and VF, PVCs, and WPW syndrome, all of which have been accurately defined and reported. Moreover, ECGi has been used to determine which patients will likely respond to CRT. Not only does the accuracy of ECGi inform its validation, but its reproducibility in many other studies also supports its reliability. These studies involved different numbers of patients and different methods, but their results were consistent. Finally, these studies reported that one of ECGi’s features was that it could provide electrical activity and excitation times along the epicardium by measuring potentials, which, in effect, could render a 3D map of the heart.^70^

## Limitations of electrocardiographic imaging

Despite being a novel diagnostic tool that is capable of providing essential information about various heart rhythm disorders non-invasively, ECGi has some limitations that should be addressed. The procedure itself is fairly complicated; it requires complex recording with hundreds of leads for a single patient, as well as dedicated and experienced personnel to obtain and interpret the ECGi findings. Regarding the number of electrodes, 125 seems to be the most suitable for capturing sensitive body-surface regions by an analysis of constructed effort gain plots. Several designed ECGi methods exceed this number, with the principle that a high number of electrodes will meet clinical requirements and improve the resolution of activation-time imaging.^71^ The application of close to 300 electrodes on the patient’s chest can be both time-consuming and inconvenient. While this problem has been improved by the implementation of a pre-constructed multi-electrode vest (*Figure 1*), it still requires added time for CT or MRI scans. Although the vest makes its application in a clinical setting easier, it too has limitations. Since it has to be fitted to different torso measurements, limited adaptability can pose a problem in the case of underweight or obese patients, or in patients with chest imperfections or deformities. The equipment itself has been reported to be uncomfortable and have limited tolerability by some patients, particularly in situations where longer recording sessions are required.^72^ Moreover, the adhesive used to fix the electrodes might irritate the skin. Further technical developments seek to facilitate easier and faster application of the equipment.^36^

The cost of the procedure is another concern. Compared to the current methods of electrophysiological assessment, ECGi can be fairly expensive for widespread use.

Imaging of epicardial potentials from the body surface requires the solution of a Laplace equation in the tissues located between the epicardium and skin, which is known as the inverse problem of electrocardiography.^73^^,^^74^ Most of the models used simplify this problem by treating the body as a uniform isotropic volume conductor, like that used in the torso-tank experiment. Inverse electrocardiography has also been applied in synthetic myocardial ischaemia localization^75^ and a macroscopic electrical heart model has been constructed using MRI-based biomechanical information to assess R- and T-wave conduction.^76^ However, this differs from the actual *in vivo* environment, where the heart is surrounded by the lungs, fat, bone and muscle tissue, each of which has its own specific conductivity. These homogeneities in conductivity cause measurement inaccuracy, resulting in the incorrect localization of electrical activity, which has been calculated to average 10 mm but can reach as high as 50 mm in some cases.^77^

One of the major discrepancies among different types of ECGi lies in the solution of the inverse problem. Although we have briefly mentioned Tikhonov regularization, this is only one of many approaches suggested by several specialists. In recent work, Karoui *et al*.^78^ compared 15 different algorithms to resolve the inverse problem using *ex vivo* and *in silico* data and found that each of them had benefits but varied according to left-ventricular, right ventricular, and bi-ventricular pacing.

Electrocardiographic imaging provides abundant and detailed information about the electrical potentials of the heart’s epicardial surface. However, the endocardial and intramural potentials differ to various degrees from those of the epicardium.^2^ For this reason, the electrical activity of structures deeper than the epicardium is far too weak and remains unrecognized by body surface ECGi.^4^ Even if signals are recorded, their localization is uncertain because the intramural potentials have different conduction paths and do not correlate with those of the epicardium. Furthermore, direct mapping of the interventricular septum, which can often be a source of ectopic impulses, is also unavailable for ECGi.^36^

The atrial wall consists of only a thin layer of myocardium, which produces very weak electrical potentials. This can sometimes pose a problem for ECGi interpretation, particularly in cases of strong ventricular activity and large QRS complexes, which then overlap P waves and make them impossible to analyse.^4^ In addition, a low signal-to-noise ratio may also be a limiting factor, because atrial potentials can easily be corrupted by artefacts and interference, making ECGi useless in the diagnosis of some ATs.^25^

In the search to construct better ECGi activation maps from unipolar ECGs for AT, it was found that the use of intrinsic deflection time as an AT marker is imprecise and leads to the appearance of false gradients. This occurs mainly because all myocardial unipolar activation signals are fragmented, or the acquisition process leads to a spatial or temporal low-pass filtering effect. To counter this limitation, the estimation of delays between neighbouring points may be a potential option to create more precise activation maps.^79^

An important drawback of ECGi, in most manufacturers, is the need to perform a CT scan to acquire a 3D image of the heart and the precise positioning of the electrodes. This exposes patients to radiation, with all of its negative effects, although the radiation dose is estimated to be minimal (approximately 148 mGy·cm).^6^ In addition, the requirement to perform a CT scan as part of the procedure limits the use of ECGi to laboratories equipped with a CT scanner. Another limitation of ECGi is the false assumption that both the heart and torso are static elements. The geometry calculations are usually based on a fixed CT image that is captured during heart diastole and breath-hold.^80^ However, during the recording of electrical potentials, these structures are inevitably in continuous motion, due to the cardiac cycle and breathing. This results in complex conduction relations that are constantly changing, thus providing room for error in inverse solution calculation.^73^

Considering that ECGi is a relatively new concept in cardiology, it is not surprising that only a few studies have examined its use in humans, as described above. Most of these studies were performed in just a few patients, and large-scale, multicentre, randomized studies will still be required to confirm the encouraging results.^39^

## Emerging technologies

A new, emerging concept seeks to develop a completely non-invasive technique for the ablation of ventricular arrhythmias. The basic idea is to combine ECGi with stereotactic body radiation therapy (SBRT), which is a method used to deliver high doses of radiation therapy to a precisely defined target, with minimal damage to the surrounding tissue.^81^ It is adopted primarily for the treatment of tumours, however, experiments suggest that it could be used as an ablation technique for cardiac arrhythmias.^82^ Cuculich *et al*.^57^ reported a case series of five patients who underwent this non-invasive approach for the treatment of VT refractory to medication. First, ECGi was performed to identify the precise location of the VT activation focus. Next, based on the acquired ECGi image, ablative radiation using SBRT was administered to the target tissue. The results showed a 99.9% relative reduction in VT occurrence in a follow-up period of 12 months.

To overcome the need for CT as part of the ECGi protocol, variants that use ultrasound and magnetic resonance imaging instead of CT are being developed.^4^ The goal is to find an imaging modality that is as accurate and readily available as CT, but without the negative effect of radiation.

Further cooperation between engineers, basic scientists and physicians is essential for discovering an optimal combination of technical capabilities, insights into the pathological mechanism, and clinical benefits of ECGi.^25^

Recently, two studies tried to use a simplified ECGi method as part of a CRT assessment.^35^^,^^38^ The system in these experiments consisted of a disposable ECG belt with only 53 electrodes that were arranged across the anterior and posterior thoracic walls. The protocol did not include any additional cardiac imaging. This simplified ECGi protocol was reported to have been useful for measuring ED before and after CRT implantation, thus helping in patient selection and optimization;^35^ and for selecting an optimal site for left ventricular stimulation.^38^

Scar after myocardial infarction (MI) represents fibrotic tissue with an altered structural architecture and pathological cellular electrophysiology with a high possibility of becoming a source for arrhythmia.^83^ In this regard, ECGi may be of particular interest in identifying and describing the electrophysiological substrate in post-MI cases. This was suggested in a study by Cuculich *et al*.,^44^ where the authors successfully characterized the MI scars of 24 patients by low voltage and fractionation; thus, complementing MRI and SPECT imaging modalities provided electrophysiological information of the scar. Importantly, scar localization determined by ECGi corresponded to that observed with MRI and SPECT. Quantification of scar substrate, although in a small cohort was also reported accurately by Wang *et al*.^60^

Algorithms can be modified for specific patient groups. As an example, a study by Potyagaylo *et al*.^52^ demonstrated the adaptation of a Fast Route algorithm with the additional application of dynamic time warping and its ability to correctly localize the origin of ectopic excitations in patients with CRT to account for local differences in conduction velocities.

In summary, current real-world clinical applications of ECGi can be seen in optimization of the CRT implantation site, identification of potential CRT responders, and more precise ablation therapies with reduced radiation exposure. Further potential uses include risk-stratification in various forms of arrhythmia. However, there is a need to validate such applications with large clinical trials. Such studies may provide robust clinical evidence that could change clinical practice in cardiology.

## Conclusion

Electrocardiographic imaging has been shown to be effective in the diagnosis of cardiac arrhythmias by further expanding the limits of the classic 12-lead ECG. The studies discussed in this review support ECGi as an effective non-invasive mapping tool which can assist in arrhythmic activation assessment and accurately locate their origins and conduction paths. Moreover, ECGi can not only localize atrial and ventricular arrhythmias but also distinguish whether they are focal or re-entrant, and whether they originate in the left or right atrium/ventricle. Electrocardiographic imaging can provide the healthcare professional with ample information that can help with the therapeutic approach, all non-invasively. Electrocardiographic imaging findings can also be combined with invasive procedures, such as ablation, resulting in higher treatment success rates. In future, as current limitations are overcome, ECGi will become more accessible and its potential further explored as a diagnosis enhancement.

## Funding

This project was supported by the Kings Health Partners, London, National Institute for Health Research (NIHR) Biomedical Research Centre and the Wellcome Trust Centre for Medical Engineering. This study was supported by Wellcome Trust (203148/Z/16/Z).

**Conflict of interest:** none declared.

### Data availability

The data underlying this article is available in Medline via PubMed at https://pubmed.ncbi.nlm.nih.gov. Moreover, the dataset was produced from reviewed articles available from sources in the public domain within the aforementioned website, with granted access from King’s College London Library Services.
